# “It only hurts when I move”: The multidimensionality of pain in nursing home residents

**DOI:** 10.1093/geroni/igaf122.2683

**Published:** 2025-12-31

**Authors:** Orah Burack, Wingyun Mak, Kreshnik Hoti, Jeff Hughes, Kimberly Bergen-Jackson

**Affiliations:** The New Jewish Home, New York, New York, United States; The New Jewish Home Research Institute on Aging, New York, New York, United States; PainChek, Sydney, New South Wales, Australia; PainChek, Sydney, New South Wales, Australia; University of Iowa, Iowa City, Iowa, United States

## Abstract

Nursing home (NH) residents often experience chronic pain and increasing cognitive deficits. In such cases effective pain assessment by staff is critical as resident self-report becomes unreliable. A consideration for staff is recognizing variability in pain due to internal and external factors. Predictable changes in pain could then be leveraged to facilitate treatment. This study examined differences in NH residents’ pain in two conditions, (1) at rest and (2) after everyday type movement. Subjects were 103 NH residents with moderate to severe cognitive impairment. Ages ranged from 64 to 107 years, 29% were males; 19% Hispanic; 54% White, 29% Black, and 17% race unknown. Ninety-five percent had chronic pain, and 85% took pain medication. Participants were assessed weekly for pain under two conditions: at rest and post-movement (walking, repositioning, or range of motion) by two raters independently, one administering the Abbey Pain Scale (APS) (0-18) and one the PainChek® tool (0-42). Higher scores indicated more pain. Order of conditions was randomly assigned per assessment. Subjects participated from one to ten weeks for a total of 552 assessments. Residents displayed significantly more pain symptoms post-movement than at rest (p < .0001) regardless of assessment tool or condition order (APS: post-movement M = 4.7 (SD = 2.91), at rest M = 2.9 (SD = 1.82); PainChek®: post-movement M = 8.2 (SD = 3.92), at-rest M = 5.8 (SD = 2.71)). Results suggest that pain levels are dynamic depending on daily activities. Determining treatment should incorporate pain assessments across different conditions. Ways to maximize the benefits of assessing pain under variable conditions will be discussed.

